# Machado-Joseph Deubiquitinases: From Cellular Functions to Potential Therapy Targets

**DOI:** 10.3389/fphar.2020.01311

**Published:** 2020-08-26

**Authors:** Chenming Zeng, Chenxi Zhao, Fujing Ge, Yuekang Li, Ji Cao, Meidan Ying, Jinjian Lu, Qiaojun He, Bo Yang, Xiaoyang Dai, Hong Zhu

**Affiliations:** ^1^Zhejiang Province Key Laboratory of Anti-Cancer Drug Research, College of Pharmaceutical Sciences, Zhejiang University, Hangzhou, China; ^2^State Key Laboratory of Quality Research in Chinese Medicine, Institute of Chinese Medical Sciences, University of Macau, Macao, Macau; ^3^Center for Drug Safety Evaluation and Research, Zhejiang University, Hangzhou, China

**Keywords:** deubiquitinase, Machado-Joseph domain-containing proteases, MJD disease, cellular functions, cancer therapy

## Abstract

Ubiquitination is known as important post-translational modification in cancer-related pathways. Human deubiquitinases (DUBs), with functions of modulating the ubiquitination process, are a family with about 100 proteins. They mainly function by cutting ubiquitin chains of the substrates. The Machado-Joseph domain-containing proteases (MJDs) is one of the sub-families of DUBs, consisting of four members, namely, Ataxin-3, Ataxin-3L, JOSD1, and JOSD2. Recent studies have provided new insights into biological functions of MJDs in the progression of Machado-Joseph disease or cancer diseases. In this review, we summarized the cellular functions and regulatory mechanisms of MJDs in Machado-Joseph disease and cancer pathways. Furthermore, we summarized MJDs genetic alterations in different human cancers by exploring the public databases (cBioportal). The aim of this review is to provide a comprehensive account based on our current knowledge about emerging insights into MJDs in physiology and disease, which might shed light on fundamental biological questions and promise to provide a potential target for therapeutic intervention.

## Highlights

• Cellular functions and regulatory mechanisms of MJDs.

• Roles and mechanisms of MJDs in Machado-joseph disease and cancer diseases.

• Ataxin-3 and Ataxin-3L are mutated in different cancers, mainly missense mutations that lead to amino acid substitutions.

• JOSD1 and JOSD2 are amplificated in several tumor types and maybe potential therapy targets.

## Introduction

During deubiquitination process, with the help of deubiquitinases (DUBs), ubiquitinated proteins can cut ubiquitins of ubiquitin-linked protein substrates and reverse the process (Amerik and Hochstrasser, 2004). It is estimated to have around 100 DUBs in human cells, consisting of seven families, including ovarian tumor proteases (OTUs), ubiquitin carboxy-terminal hydrolases (UCHs), ubiquitin-specific proteases (USPs), Machado-Joseph domain-containing proteases (MJDs), JAMM/MPN domain-associated metallopeptidases (JAMMs), monocyte chemotactic protein-induced protein (MCPIP), and Zinc finger with UFM1-specific peptidase domain protein (ZUFSP). (Nijman et al., 2005; Reyes-Turcu et al., 2009; Kwasna et al., 2018).

**Figure 1:**
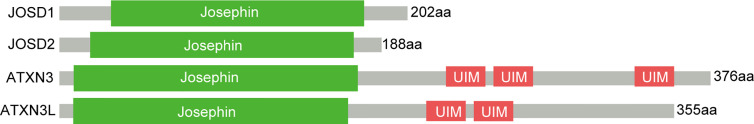
Overview of MJDs domain structures. In addition to the Josephin domain, Ataxin3 has three UIMs flanking a polyglutamine repeat.

MJDs is the smallest family of the DUBs. All MJD family members have a common cysteine protease domain, namely Josephin domain (**Figure 1**), consisting of around 180 amino acids. Ataxin-3 and the Ataxin-3-like protein (Ataxin-3L) are two Josephin proteins in human cells, both of which consists of a single Josephin domain at their N-terminus with a flexible C-terminal domain of comparable length. While JOSD1 and JOSD2 only contain a single Josephin domain, throughout all eukaryotes, Josephin proteins are highly conserved. At the same time, homologs of Josephin proteins, which can be found in plants and protozoans, are widely distributed among metazoans. Although this highly conserved domain actually implies important roles of the MJD family members, in most cases, their biological functions remain largely unanswered.

Accumulating evidence indicates that DUBs, with the function of driving the tumor progressions, resembling to cancer stem cells and *etc* (Tang et al., 2020), are now emerging as attractive targets for the development of novel cancer therapies (Harrigan et al., 2018). The elucidation of physiological and pathophysiological roles of DUBs may contribute the current interest in DUBs as therapeutic targets.

In this article, we first review the cellular functions of MJD family members by highlighting their relevance in MJD. Then, we focus on increasingly recognized involvement of MJDs in human cancers and further discuss new findings revolving the prospect of MJDs as a target in human cancer therapy.

## Deubiquitinating Activity of MJDs

Ataxin-3 contains an N-terminal Josephin domain (JD), a conserved module named after the Machado-Joseph disease, two ubiquitin interacting motifs (UIMs), a polyQ stretch, and a flexible variable tail. The C-terminal region ends with a third UIM in one splice variant. Like a poly-ubiquitin chain-cutting enzyme, Ataxin-3 has deubiquitinating activity and functions to cleave ubiquitin chains. Deubiquitinating activity of Ataxin-3 is abrogated by a cysteine (C14) mutation in the catalytic site (Burnett et al., 2003). Ataxin-3 is differentiated from other deubiquitinating enzymes by these properties (Mao et al., 2005). *In vitro* studies show that both Ataxin-3 and Ataxin-3L can cleave K48 and K63 linked chains, though enzyme activity in this assay is inadequate when using shorter chain lengths (≤6).

Full-length Ataxin-3L consists of a Joesephin Domain at N-terminal, two motifs interacting with ubiquitin (UIM’s) and a C-terminus composed of a polyglutamine. Though Ataxin-3L demonstrates a higher deubiquitinating activity compared with that of Ataxin-3, Ataxin-3L, and Ataxin-3 possess 85% sequence identity (Weeks et al., 2011). The substantial difference in DUB activities for Ataxin-3 and Ataxin-3L is interesting considering their highly sequence identity in the Josephin domains. Ataxin-3L’s crystal structure of Josephin domain and ubiquitin complex shows that the ubiquitin substrates of Ataxin-3 and Ataxin-3L have different binding modes while having large levels of sequence and structure resemblance. Thus, they either have distinct ubiquitin binding points along the catalytic pathway or have different means of substrate recognition mechanisms. It can be seen that Ataxin-3L is much more active than Ataxin-3. However, it can also be concluded that the Ataxin-3’s DUB activity is restrained to remain low as three mutations of amino acid residues (cysteine14, histidine119, and asparagine134) in Ataxin-3 can almost equalize their deubiquitinase activities. The activating mutations may also change the preferences of ubiquitin binding modes.

JOSD1 and JOSD2 have similar Josephin domain and share a high similarity in amino acid sequence. They diametrically differ in some other aspects, such as basal deubiquitinating activity *in vitro*, the *in vitro* capacity for ubiquitination and localizations at subcellular level. JOSD1 functions to cleave ubiquitin chains only when it is monoubiquitinated (Seki et al., 2013). Furthermore, the deubiquitinating activity of JOSD1 would be lost upon a cysteine (C36) mutation in the catalytic site (Seki et al., 2013). JOSD2 does not need to be ubiquitinated before it is endowed with catalytic activity, at least at *in vitro* system (Seki et al., 2013). And the mutation of a cysteine (C24) in the catalytic site will result in the reduction of deubiquitinating activity of JOSD2. A recent study also shows that JOSD2 preferentially cleaves K11, K48 and K63 linked ubiquitin conjugates, revealing its potential in protein control maintenance (Grasty et al., 2019).

## Cellular Functions of MJDs

Among the four MJD members, the best characterized one is Ataxin-3. Ataxin-3 has a tendency for K63 linked ubiquitin chains (Todi et al., 2009b). Similar to JOSD1, catalytic activity of Ataxin-3 can be regulated by ubiquitylation (Todi et al., 2009a). Ataxin-3 is related to two cellular processes: transcription repression (Kawaguchi et al., 1994) and protein homeostasis (Reina et al., 2010). Ataxin-3 interacts with a variety of transcription regulators like TAFII130 (Shimohata et al., 2000), cAMP response element binding protein (CREB)-binding protein (CBP) (Chai et al., 2002), RAD23 (Wang et al., 2000), NCoR, HDAC3 and HDAC6 (Burnett and Pittman, 2005; Evert et al., 2006) (**Table 1**). The nuclear localization of Ataxin-3 has also been proved to be necessary in the symptoms in spinocerebellar ataxia type 3 (SCA3) *in vivo* (Bichelmeier et al., 2007).

**Table 1:** Cellular functions of MJDs and mechanisms.

MJDs	Cellular functions	Substrates and mechanisms	Disease and cancer	Reference
Ataxin-3	Represses transcription expression	TAFII130, CBP, RAD23 NCoR, HDAC3, and HDAC6	Neurodegenerative disease	(Shimohata et al., 2000; Wang et al., 2000; Chai et al., 2002; Burnett et al., 2003; Evert et al., 2006)
	Maintains E3 ligase protein expression	CHIP and parkin	Neurodegenerative disease	(Connell et al., 2001; Durcan and Fon, 2011; Scaglione et al., 2011; Durcan et al., 2012; Bai et al., 2013)
	Activates AKT/mTOR pathway	Inhibits the expression of PTEN	Testicular cancer	(Shi et al., 2018)
	Decreases cell viability and migration	Expression suppressed by miR-25	Colon cancer	(Krauss et al., 2019)
Ataxin-3L	Promotes cell proliferation, and tumorigenesis	Upregulates KLF5 protein	Breast cancer	(Buus et al., 2009)
JOSD1	Promotes drive acquired chemoresistance	Upregulates MCL1 protein	Gynaecological cancer	(Wu et al., 2019)

Biological functions ascribed to Ataxin-3L remain largely unknown. Recent study shows that the Ataxin-3L has been identified as the DUB of Kruppel-Like Factor 5 (KLF5) (Buus et al., 2009). Ataxin-3L binds to KLF5, and remove the ubiquitin chains on KLF5, thus preventing its degradation. KLF5 functions as a transcriptional factor essential for the promotion of breast cell proliferation, survival, and tumorigenesis. According to this study, Ataxin-3L would antagonize ubiquitination and proteasome-mediated degradation of KLF5, which means this DUB’s inhibiting activity may be a potential therapeutic target for breast cancer intervention.

Myeloid cell leukaemia 1 (MCL1) is a critical antiapoptotic member of the BCL-2 family, which is thought to be promising therapeutic strategy for overcoming chemoresistance (Cory and Adams, 2002; Delbridge et al., 2016). JOSD1 could upregulate MCL1 protein by acting as a deubiquitinase and then cause chemo-resistance in gynaecological cancer by inhibiting mitochondrial apoptotic signaling *via* MCL1 stabilization (Wu et al., 2019). In addition, JOSD1 is membrane-associated, and it can affect cellular motility and endocytosis (Seki et al., 2013). JOSD1 predominantly localizes at the plasma membrane. However, it does not have any hydrophobic motif to maintain the membrane localization, thus it must be targeted to plasma membrane through interactions with membrane proteins or lipids. In the contrast, while sharing highly similar amino acid sequence with JOSD1, JOSD2 is localized mainly in the cytosol, but not on the cell membrane. Notably, JOSD1’s ubiquitination can regulate its catalytic activity, but this modification is not necessary for JOSD2 to exert its DUB function. These findings implicate that, although with high similarity in amino acid sequences, these two MJD members are endowed with differential interacting partners, regulated by diverse mechanisms, and involved in different cellular functions.

## Regulatory Mechanisms of MJDs

The regulatory mechanisms of most MJD family members are poorly understood. A recent study shows that Ataxin-3 promoter’s DNA methylation may contribute to instability of AAO and CAG amino acid sequence repeats in SCA3/MJD, which elucidates a new xenogeneic explanation of SCA3/MJD disease (Wang et al., 2017). If the CAG repeats in stability and genetic anticipation of SCA3/MJD proves to be determined by the epigenetic change, ultimately epigenetic therapy may become possible to control repeats stability and to delay the AAO for the SCA3/MJD, and the potentiality for the epigenetic-based therapy may extend to other repeats-associated neurodegenerative diseases as well. Besides, family members of miR-181 and miR-25 can bind to the 3’UTR to inhibit Ataxin-3’s expression (Krauss et al., 2019). The results also show the cell type-specific protection mechanism since miRNAs have well-defined developmental and cell-type expression patters. Elevated expression of miR-181 and miR-25 family members in SCA3LCs can reduce cytotoxic effects mediated by polyQ. Therefore, when it comes to the therapy of SCA3, miRNA mimics targeting miR-25 and miR-181 subfamilies may be a promising strategy. Ataxin-3 also restricts transcription of PTEN in lung cancers (Sacco et al., 2014). Though lung cancer cells (A549) treated with vorinostat (SAHA), a broad HDAC inhibitor, only shows limited response, PTEN induction is enhanced with Ataxin-3 depletion significantly. These interventions also extremely decrease cell viability. Therefore, Ataxin-3 presents as an independent and complementary therapy target with downregulation expression of PTEN in cancers. Further studies are required to elucidate the regulatory mechanisms of Ataxin-3 and other MJD family members.

## Crystal Structure of MJDs

The crystal structure of Josephin domain of Ataxin-3 shows that fragment at carboxyl-terminal has 14 glutamine residues with irregular coil and α-helical conformations. The polyQ sequence in α-helical structure is stabilized by intra-helical hydrogen bonds mediated by glutamine side chains. The intra-helical hydrogen-bond interactions in the side chains of glutamine on the polyglutamine (poly Q) α-helix stabilize the secondary structure (Zhemkov et al., 2016). The analysis information obtained from Ataxin-3 structure of its polyQ domain shows that when glutamines interact with intra-helical hydrogen, it can result in aggregation, which may be the basis of the pathogenic explanation of polyQ-expansion proteins. The Josephin domain structure is identical in Ataxin-3L and Ataxin-3 (Weeks et al., 2011).

In contrast to Ataxin-3 and Ataxin-3L, JOSD1 and JOSD2 have received less attention, not only on the cellular functions as well as protein substrates, but also in the crystal structures. A very recent research depicted the crystal structure of JOSD2 and its substrate specificity. Several large disordered loops are seen in the structure, suggesting that JOSD2 might be a highly dynamic enzyme. JOSD2 lacks several allosteric sites which were found in Ataxin-3, but its structure suggests potential regulation *via* ubiquitination of a loop adjoining the active site (Grasty et al., 2019). Additionally, in the covalent complex of JOSD2 and ubiquitin, the ubiquitin molecule is covalently attached to cysteine-24, which moves side chain of this residue away from histine-125 and toward the binding site of the substrate. Hence, this structure represents the species formed post-nucleophilic attack, rather than the pre-catalysis species, in which cysteine-24 would be expected to interact with histine-125. Therefore, we can conclude from this structure that histine-125 might also be important for the deubiquitinating activity and biological functions of JOSD2.

## Ataxin-3 in Machado-Joseph Disease

MJDs have been implicated to be closely related to neurological disorders such as Machado-Joseph disease (MJD), also known as spinocerebellar ataxia type 3 (SCA3) (Haberhausen et al., 1995), which is thought as the most usual form of spinocerebellar ataxia (Haberhausen et al., 1995). Resulted by the abnormal extension of CAG trinucleotides present in the coding regions of single or unrelated genes, Machado-Joseph disease is one of the polyglutamine (polyQ) diseases, which encompass nine inherited neurodegenerative disorders (Matos et al., 2019). Proteolytic liberation of highly aggregation-prone polyQ fragments from the protective sequence of Ataxin-3 has been proposed to trigger the formation of Ataxin-3-containing aggregates, the neuropathological hallmark of Machado-Joseph disease.

As a therapeutic target in neurodegenerative disease, Ataxin-3 has been well characterized in the progression of Machado-Joseph disease. Throughout peripheral and neuronal tissues, Ataxin-3 is broadly expressed in different cell types (Paulson et al., 1997). Ataxin-3 fragments can be found in brain tissues of Machado-Joseph disease patients and mice expressing mutant Ataxin-3 (Q71) (Goti et al., 2004). Recent research suggested that, through cutting the substrates-linked polyubiquitin chains before the digestion, Ataxin-3 can help the proteasome to degrade ubiquitinated proteins. Ataxin-3 can also bind K48 and K63 linked polyubiquitin chains disregarding the length of the polyQ (Chai et al., 2004; Winborn et al., 2008; Carvalho et al., 2018).

Ataxin-3 interacts with two E3 ligases that are essential for maintaining normal cellular hemostasis, namely C terminus of Hsc70-interacting protein (CHIP) and parkin. CHIP is a neuroprotective E3 implicated in protein quality control by interacting with chaperones to promote the degradation of misfolded proteins (Connell et al., 2001). Ataxin-3 and Ube2w coordinately regulate the ability of CHIP to ubiquitinate itself (Scaglione et al., 2011). Mechanically, Ube2W promotes the monoubiquitination of CHIP and thus stabilizes the interaction between CHIP and Ataxin-3. Ataxin-3 not only deubiquitinates CHIP, but also trims polyubiquitin chains on CHIP substrates, thereby regulating the length of ubiquitin chains. Furthermore, PolyQ expanded ataxin-3 binds tighter to CHIP, and CHIP levels are lower in the brains of MJD transgenic mice, indicating that loss of one or both E3 partners may be a contributing factor in the progression of SCA3. In addition to CHIP, Ataxin-3 also interacts with another E3 ligase, which is parkin (Durcan et al., 2012; Bai et al., 2013). Through the interaction with parkin, Ataxin-3 regulates the ability of parkin to ubiquitinate itself, with Ataxin-3 decreasing parkin self-ubiquitination. Moreover, mutant type Ataxin-3 promotes the clearance of parkin *via* the autophagic degradation of parkin (Durcan and Fon, 2011), raising the possibility that elevated turnover of parkin may be the partial reason of Machado-Joseph disease.

As for the other family members of MJD, Ataxin-3L, JOSD1, and JOSD2, no clues have indicated their roles in MJD. Nevertheless, there is emerging evidence indicating that Ataxin-3, Ataxin-3L, JOSD1, and JOSD2 are also implicated in cancer progression.

## MJDs in Cancer-Related Pathways

Altered DUB activity is associated with a multitude of pathologies including carcinogenesis and cancer malignancy. Mounting evidence shows that the aberrant expression or mutations of DUBs may promote the development of malignant cancer. Therefore, DUBs represent novel candidates for target-directed drug development.

Recent research showed that non-small-cell lung cancer is related to Ataxin-3, so are testicular cancer and human colon cancer. In Ataxin-3-depleted non-small-cell lung cancer, mRNAs of PTEN and PTENP1 decay rapidly with transcriptional inhibition as Ataxin-3 acts primarily by repressing their transcription (Sacco et al., 2014). PTEN is often silenced in non-genomic mechanisms in cancer. Thus, minor variations in PTEN expression can affect prognosis. Therefore, the reactivation of PTEN may be one promising treatment strategy. In this case, combination therapy, with Ataxin-3 inhibitors and HDAC inhibitors, is a realistic strategy for PTEN-inhibited lung cancers. In testicular cancer, Ataxin-3 overexpression can promote cell multiplication. Also, up-regulated Ataxin-3 is able to activate the AKT/mTOR signaling pathway and repress PTEN expression (Shi et al., 2018). These results may make the mechanism of testicular cancer easier to comprehend, as Ataxin-3 exerts its effects by suppressing the PTEN expression and indirectly activating the Akt/mTOR signaling pathway. They demonstrate how Ataxin-3 can be both a promising biomarker and therapeutic target for testicular cancer. As for colon cancer, it has been reported that miR-25 promotes cell growth, cell migration, and inhibits apoptosis *via* Ataxin-3 expression. The miR-25/Ataxin-3 axis provides a novel insight into the pathogenesis of human colon cancer, particularly with respect to promote proliferation and metastasis of colon cancer, and Ataxin-3 represents a potential therapeutic target for human colon cancer (Li et al., 2019). Intriguingly, Ataxin-3 protein expression in gastric cancer tissues was significantly correlated with the expression of mutated p53, suggesting that Ataxin-3 may also be related to the role of p53 in the oncogenic process of gastric cancer (Zeng et al., 2014). We therefore speculate that expression of Ataxin-3 can enhance the malignant features of cells by promoting the expression of mutant p53, such as in the carcinogenesis of gastric cancer. Therefore, Ataxin-3 may serve as a potential intervention target for gastric cancer treatment. Importantly, further research on Ataxin-3 may offer a more comprehensive understanding of Ataxin-3 and might uncover the potential biomarkers for the progression of gastric cancer. Increasing evidence also suggests roles for Ataxin-3 in promoting DNA damage repair. Ataxin-3 functions as DUB to stabilize early targets of ataxia telangiectasia mutated (ATM) and ataxia telangiectasia and Rad3-related protein (ATR) coupled DDR pathways to promote DNA repair and checkpoint signaling (Smith et al., 2010; Marechal and Zou, 2013). Ataxin-3 interacts with DNA end-processing enzyme polynucleotide kinase 3’-phosphatase (PNKP) to promotes PNKP’s phosphatase and DNA repair activities (Chatterjee et al., 2015; Gao et al., 2015). Ataxin-3 also stabilizes the checkpoint kinase 1 (Chk1) and tumor protein 53 (p53), both of which are critical for DNA repair (Liu et al., 2016; Tu et al., 2017).

The influence of Ataxin-3L on cancer-related signaling pathways has not been well characterized. Buss R et al. found that Ataxin-3L promotes the migration of NSCLC cells, yet the mechanism remains unclear (Buus et al., 2009). Recently, a genome-wide siRNA screening identified Ataxin-3L as a KLF5 DUB (Ge et al., 2015). Ataxin-3L interacts with the KLF5 and reverts the polyubiquitination on KLF5 protein. Besides, knockout of Ataxin-3L inhibits the proliferation of breast cancer cells partly through KLF5. Furthermore, high level of Ataxin-3L are associated with poor overall survival of breast cancer patients. Therefore, Ataxin-3L is a novel positive regulator of KLF5 and may serve as potential therapeutic target for breast cancer treatment.

Recent studies suggest JOSD1 as one of the most upregulated DUBs during the development of chemo-resistance in gynaecological cancer (Wu et al., 2019). Mechanistically, JOSD1 can cleave the K48 ubiquitin chains linked on MCL1 to upregulate MCL1 protein. JOSD1 inhibits the mitochondrial apoptosis pathway and exerts anti-apoptosis effects and results in chemo-resistance in gynaecological cancer by stabilizing MCL1. Also, patients with poor ovarian cancer prognosis have high JOSD1 expression. This may explain the reason why serum JOSD1 levels can be helpful for clinical diagnosis (Wu et al., 2019). As a result, JOSD1 is an ideal therapeutic target as well as a diagnosis indicator.

## MJDs Alterations in Cancer

**Figure 2:**
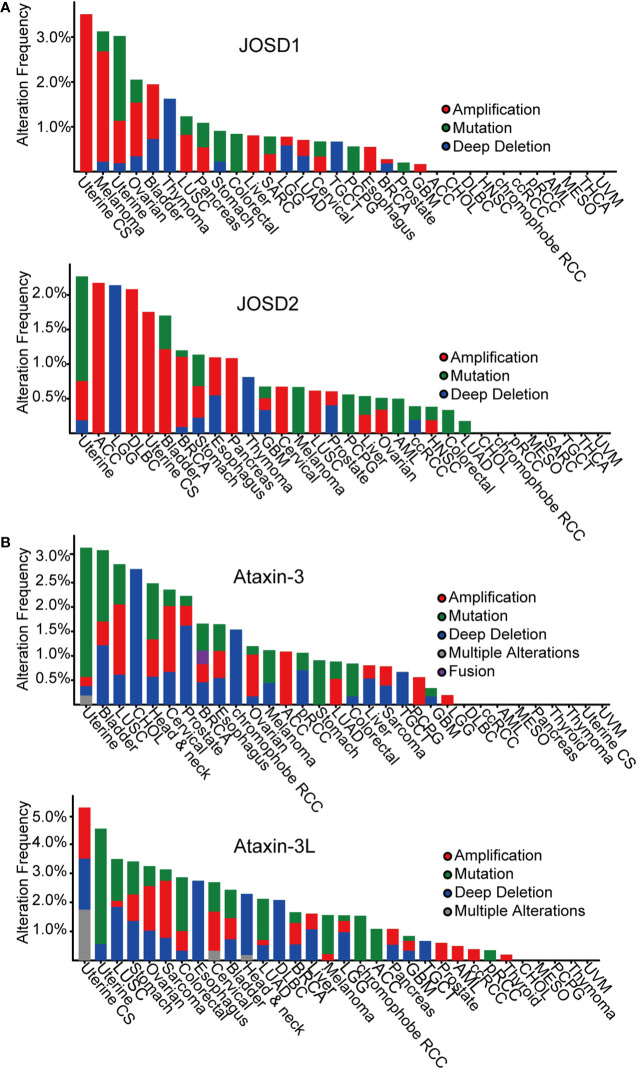
Pan-cancer analysis of MJDs genomic alterations in cBioportal database. **(A)** JOSD1 and JOSD2 are amplified in most human cancers. **(B)** Ataxin-3 and Ataxin-3L are mutated or lost in most human cancers.

According to The Cancer Genome Atlas (TCGA) cBioportal, analysis of MJD genetic aberrations in cancers, uncovers that genetic alterations of the MJD family members are associated with cancer (**Figure 2**, **Table 2**). The most common alterations found in cancers are mutation and amplification.

**Table 2:** Pan-cancer analysis of MJDs genomic alterations.

MJDs	Amplification	Mutation	Deep deletion
Ataxin-3	LUSC Head & neck Cervical ACC	Uterine Bladder LUSC Head & neck Stomach Colorectal	Bladder LUSC CHOL Cervical Prostate chromophobe RCC
Ataxin-3L	Uterine CS Ovarian Sarcoma Cervical	Uterine LUSC Stomach Colorectal LUAD Melanoma chromophobe RCC ACC	Uterine CS LUSC Stomach Esophagus Head & neck DLBC Liver LGG
JOSD1	Uterine CS Melanoma Uterine Ovarian Bladder LUSC Pancreas Liver Esophagus	Uterine Stomach Colorectal PCPG	Uterine Bladder Thymoma LGG TGCT
JOSD2	Uterine AGC DLBC Uterine CS Bladder BRCA Pancreas Cervical LUSC	Melanoma PCPG AML Colorectal	LGG Esophagus Thymoma

Analysis has found loss-of-function mutations and deep deletions of Ataxin-3 as well as Ataxin-3L in many cancers, such as uterine, bladder, lung, and cholangiocarcinoma cancer. The most frequent alteration in Ataxin-3 and Ataxin-3L is mutation in different cancers, mainly missense mutations that lead to amino acid substitutions.

In contrast, mutational alteration of JOSD1 and JOSD2 in cancer is less common. Nevertheless, both JOSD1 and JOSD2 expression have been found to be significantly alterated in several tumor types. Amplification is the most common finding in various cancers. It is particularly frequent in uterine, melanoma, ovarian, and bladder cancer for JOSD1. As for JOSD2, the amplification is more frequent in a range of tumors including uterine, adrenocortical carcinoma (ACC), lymphoid neoplasm diffuse large B-cell lymphoma (DLBC), bladder, breast cancer, stomach cancer, and pancreas. These gain-of-function amplifications of JOSD1 and JOSD2 are thought to cause elevated expression to promote their tumor-promoting functions.

## Conclusion and Future Perspective

As a family member of MJDs, Ataxin-3 has been well characterized to be involved in the progression of Machado-Joseph disease. The rest family members such as Ataxin-3L, JOSD1, and JOSD2 have been identified to be associated with tumor progression recently. In the future, more research should focus on the correlation between clinical samples and MJDs. Overexpressed in multiple cancers, JOSD1 and JOSD2 might serve as oncogene factors to promote cancer development. Hence, it is of great significance to target JOSD1 and JOSD2 in hyper-activated cancers with small molecular inhibitors. Furthermore, more studies are required to uncover the protein structures of MJDs as to develop potential inhibitors for the treatment of MJDs-related cancer.

## Author Contributions

HZ and XD conceived, designed the conception of review article, and made the amendments of the paper. CZe conducted the paper. FG, YL, JC, MY, JL, QH, and BY collected the related research articles. All authors contributed to the article and approved the submitted version.

## Conflict of Interest

The authors declare that the research was conducted in the absence of any commercial or financial relationships that could be construed as a potential conflict of interest.
